# Asthma Control and Asthma Medication Use among Swedish Elite Endurance Athletes

**DOI:** 10.1155/2018/4646852

**Published:** 2018-03-18

**Authors:** Hampus Persson, Anne Lindberg, Nikolai Stenfors

**Affiliations:** Department of Public Health and Clinical Medicine, Division of Medicine, Umeå University, Östersund, Umeå, Sweden

## Abstract

**Background:**

Asthma is common in elite athletes. In this study, we examined the use of asthma medication and asthma control in endurance athletes in Sweden and compared the findings with those in a reference group of patients with asthma.

**Methods:**

The Asthma Control Test (ACT) and a questionnaire on asthma, respiratory symptoms, and medication use were posted to endurance athletes (*n*=711) and the reference group of patients with asthma (*n*=1026). Four hundred and sixty-nine athletes (66%) responded, of whom 141 (20%) reported physician-diagnosed asthma. In the reference group, 397 (39%) responded.

**Results:**

Seventy-seven percent of the athletes with asthma reported using asthma medication during the previous year; 39% used short/long-acting *β*2-agonists, 31% used inhaled corticosteroids, and 31% used both daily. According to the ACT scores, 19%, 24%, and 58% of athletes with asthma had uncontrolled, partially controlled, or well-controlled asthma, respectively. After adjustment, there was no difference in ACT scores or daily use of asthma medication between the study groups.

**Conclusions:**

Many endurance athletes had uncontrolled or partially controlled asthma, and one-third used inhaled corticosteroids and long-acting *β*2-agonists daily. Their adjusted ACT scores and use of asthma medication were similar to the values in the reference population.

## 1. Introduction

The prevalence of asthma in Sweden is estimated to be 10% in the population aged 16–35 years [1]. However, the prevalence of asthma is higher in athletes, especially those participating in sports associated with high ventilation rates [2–5]. Recently, the prevalence of physician-diagnosed asthma among Swedish elite cross-country skiers was estimated at 35% [6].

The reason for the increased prevalence of asthma in winter endurance athletes is believed to be a consequence of repeated and prolonged inhalation of dry air, leading to heat loss, dehydration, and airway damage. In addition, the cooling process seems to induce bronchoconstriction via the parasympathetic nervous system, and subsequent rewarming leads to mucosal edema and further narrowing of the airways [7, 8].

According to the guidelines, the first step in the treatment of asthma is a short-acting *β*2-agonist (SABA), as required. Step 2 is the addition of regular daily inhalation of corticosteroids (ICS). In Step 3, the dose of ICS can be increased and/or a daily long-acting *β*2-agonist (LABA) can be added [9]. A recent population-based study in Sweden showed that 19% of patients with asthma regularly used the third step of asthma treatment, that is, a fixed combination of ICS + LABA [10]. In the western parts of Sweden, the use of pharmacotherapy for asthma increased by 54% between 1992 and 2010, and the proportion of subjects with asthma using ICS increased by fivefold during that time [10].

A significant increase in use of asthma medication was documented among Finnish Olympic athletes between 2002 and 2009, even though there was no significant increase in the prevalence of either asthma or asthma symptoms, and 4% used ICS + LABA [11]. It was reported that 7% of Danish elite athletes used asthma medication in 2005 and that 79% used ICS alone or in combination with SABA or LABA [12]. Another study from Australia found no difference in the prevalence of asthma or use of *β*2-agonists between athletes and an age-matched control group [13]. In contrast, Finnish athletes competing in endurance and team sports were reported to use significantly more asthma medication than an age-matched control group [14]. However, none of these studies reported on the use of asthma medication in relation to asthma control in athletes.

Selection of appropriate asthma medication is a key to disease control, and there is a lack of data on asthma control in elite endurance athletes. The primary aims of this study were to examine the level of asthma control and to determine the use of asthma medication in detail, specifically SABA/LABA, ICS, and ICS + LABA, among elite endurance athletes with current asthma. A secondary aim was to compare these findings with those in a reference population of patients with asthma recruited from health care registers.

## 2. Materials and Methods

### 2.1. Study Design

This cross-sectional survey was part of a 5-year prospective postal questionnaire study on the prevalence, incidence, and remission of airway symptoms and asthma in Swedish elite endurance athletes. The study has been described in detail previously [6] and was approved by the Regional Ethical Review Board at Umeå University, 2011-186-31M (2011-06-07) and 2016/146-32 (2016-05-03).

### 2.2. Settings and Participants

Two study populations were included. One study group included Swedish elite athletes participating in cross-country skiing, biathlons, ski-orienteering, or orienteering at international or highest national levels. These athletes consisted of students at seven National Elite Sports Schools, Swedish junior and senior national team members, students at the three Swedish Ski Universities where elite athletes combine an elite career with higher education, and the top 80 Swedish orienteers aged 20–30 years according to the national ranking in 2011. A total of 711 eligible athletes were invited to participate in a postal questionnaire survey in September 2014 and 2015. The other study (reference) group included patients who were identified through primary and specialist health care records to have been born between 1985 and 1999 in the region of Jämtland-Härjedalen and to have a diagnosis of asthma (ICD code J45) in 2011–2015. Patients with asthma who were already included in the group of athletes were excluded. In total, 1026 eligible reference subjects were invited to participate in the postal questionnaire survey in September 2016. To match the smoking habits of the athletes, ex-smokers and current smokers were excluded from the analyses.

### 2.3. Study Variables

The postal questionnaire was a shortened version of the European Community Respiratory Health Survey II, which includes questions on asthma, drug utilization, airway symptoms, smoking history, family history of asthma, and health care contacts [15], and included an additional question regarding physical training. Subjects with physician-diagnosed asthma were also asked to complete the Asthma Control Test (ACT). The ACT is a well-validated questionnaire consisting of 5 questions on activity limitations, shortness of breath, awakenings with asthma, use of rescue medication, and general asthma control [16]. One reminder was sent out to all nonresponders.

The key variables investigated in the study are outlined below, along with an explanation of how each variable was assessed:

1. “Physician-diagnosed asthma” was identified by the responses to the questions “Have you ever had asthma?” and “Was it diagnosed by a doctor?”
2. “Medication” was identified by the responses to the question, “Have you used any asthma medication, including inhalers, sprays, or pills, during the last 12 months?” Asthma medication was categorized as SABA/LABA, ICS, or ICS + LABA. Montelukast was used by a small proportion in both study groups and is not presented in the results. The frequency of use of asthma medication was categorized as “never,” “sometimes,” “for more/less than 2 months per year,” or “daily.”
3. “Current asthma” was defined as physician-diagnosed asthma and use of asthma medication during the previous 12 months.
4. “Age of asthma onset” was determined by the response to the question, “How old were you when you had your first asthma attack?”
5. “Smoking status” was determined by the responses to the questions, “Have you been smoking one or more cigarettes per day for at least one year?” and “Have you been smoking during the last month?”
6. “Training” was assessed by the response to the question, “On average, during the last 12 months, for how many hours/week did you exercise so much that you got out of breath or became sweaty?”
7. “Family history of asthma” was determined by the response to the question, “Do any of your parents or siblings have asthma?”
8. “Allergy” was identified from the response to the question, “Do you have any nasal allergies, including hay fever?”
9. “Health care contact” was assessed by the response to the question, “Have you had any health care contacts due to airway problems in the last 12 months?”
10. “Asthma control” was assessed using the ACT and was reported both as a continuous variable ranging from 5 to 25 points and was categorized as “uncontrolled” (≤19 points), “partially controlled” (20–21 points), or “well controlled” (≥22 points) [16].

### 2.4. Statistical Analysis

The assumption was made a priori that 25% of the athletes and 15% of the reference subjects would be using ICS + LABA daily. We calculated that inclusion of 146 athletes would require 294 reference subjects to reach a power of 80% with an *α* level of 5%. A reference group of 1000 patients with a response rate of 30% was thus expected to be sufficient to detect a difference in daily use of ICS + LABA between the study groups.

Reference subjects who answered “No” (*n*=11) or had no medication recorded or missing data regarding “physician-diagnosed asthma*”*(*n*=21) were excluded. The two groups were matched with regard to smoking habits in order to exclude smoking as a source of bias in the reference group; ex-smokers and current smokers (*n*=90) were excluded from the reference group. Use of asthma medication and the ACT score data are presented only for subjects with current asthma.

Continuous variables were compared between the study groups using Student's *t*-test and categorical variables using Pearson's chi-squared test. To compare the use of asthma medication between the study groups, the subjects were dichotomized, respectively, into those who did and did not use SABA/LABA, ICS, or ICS + LABA on a daily basis. Similarly, the proportions of subjects with “uncontrolled,” “partially controlled,” or “well-controlled” asthma were compared between the study groups on the basis of the dichotomized responses on the ACT. A *p* value < 0.05 was considered to be statistically significant.

After the bivariate analysis, multivariate Poisson regression analyses were performed in each group, and the data for the two study groups were then pooled to test for an independent association between the ACT score and being an elite athlete. Subject's age, age at asthma onset, allergy, shortness of breath after exercise, and respiratory-related health care contacts during the previous 12 months were included as covariates in the model.

## 3. Results

### 3.1. Subject Characteristics

**Figure 1:**
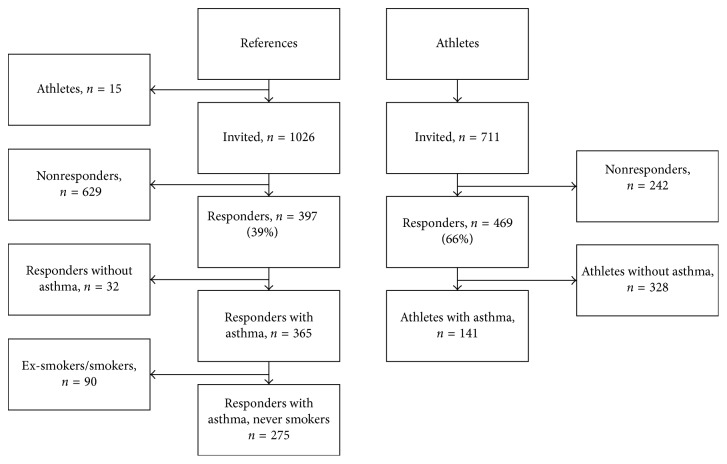
Flowchart of study participants.

Four hundred and sixty-nine (66%) of the 711 athletes invited to participate in the survey responded, and all were nonsmokers. Of these, 141 (20%) reported having physician-diagnosed asthma. Three hundred and ninety-seven (39%) of the 1026 eligible reference subjects responded. Of these, 122 were excluded either because they did not have physician-diagnosed asthma (*n*=32) or were ex-smokers or current smokers (*n*=90), leaving 275 reference subjects for inclusion in the analysis (Figure 1). Among the athletes with asthma, 82 (58%) were cross-country skiers, 26 (18%) were biathletes, and 19 (13%) were orienteers. Eighty-six (54%) were or had been national team members, and 55 (39%) were secondary school students.

A detailed description of the two study populations is presented in Table 1. The athletes had a lower mean age and a higher mean number of training hours per week when compared with the reference group. The reported age of asthma onset was older in the athletes than in the reference subjects. The prevalence of current physician-diagnosed asthma, determined by the use of asthma medication during the previous 12 months, was lower in the athlete group than in the reference group (77% versus 88%, *p*=0.002).

### 3.2. Asthma Control

When classified by ACT scores, 58% of the athletes with current asthma reported that their asthma was well controlled, whereas 23% reported partially controlled asthma and 19% reported uncontrolled asthma (Table 2). These three subgroups of athletes, based on the level of asthma control, differed in their number of health care contacts during the previous 12 months, daily use of asthma medication, and use of SABA/LABA and ICS + LABA; the highest numbers in all categories were for athletes reporting uncontrolled asthma (Table 3).

In the reference group, 102 patients (42%) reported well-controlled asthma (Table 3). Those with uncontrolled asthma had more shortness of breath, used more daily asthma medication, and had had more health care contacts during the previous 12 months.

**Figure 2:**
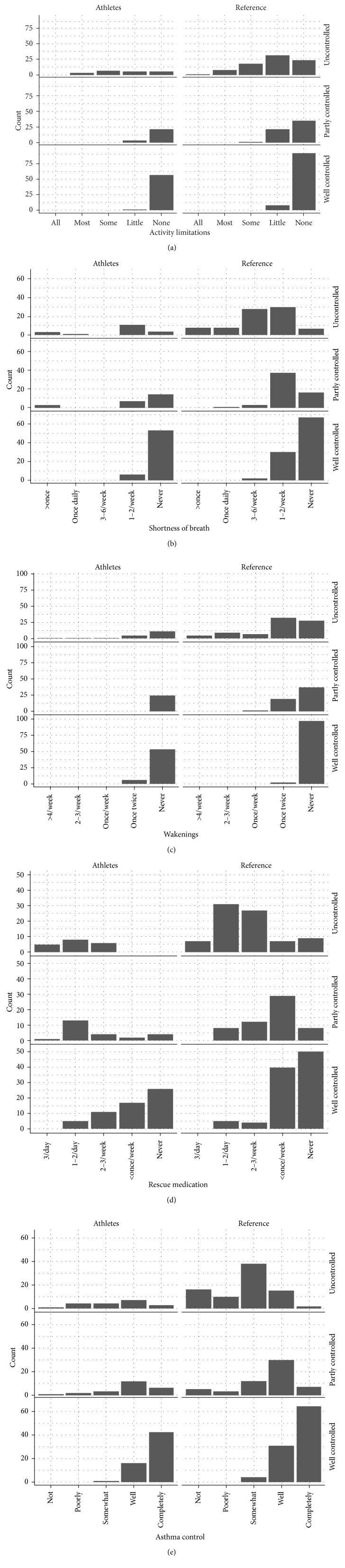
Results for each ACT question. The results are stratified by study population and ACT category. (a) “In the past 4 weeks, how much of the time did your asthma keep you from getting as much done at work, school or at home?” (all of the time, most of the time, some of the time, a little of the time, or none of the time). (b) “During the past 4 weeks, how often have you had shortness of breath?” (more than once a day, once a day, 3 to 6 times a week, once or twice a week, or not at all). (c) “During the past 4 weeks, how often did your asthma symptoms (wheezing, coughing, shortness of breath, chest tightness, or pain) wake you up at night, or earlier than usual in the morning?” (4 or more nights a week, 2 to 3 nights a week, once a week, once or twice, or not at all). (d) “During the past 4 weeks, how often have you used your rescue inhaler or nebulizer medication?” (3 or more times per day, 1 or 2 times per day, 2 or 3 times per week, once a week or less, or not at all). (e) “How would you rate your asthma control during the past weeks?” (not controlled at all, poorly controlled, somewhat controlled, well controlled, or completely controlled).

Athletes whose ACT scores indicated well-controlled asthma reported no or little limitation in daily activities (Figure 2(a)), seldom experiencing shortness of breath (Figure 2(b)), and few awakenings with asthma (Figure 2(c)), and considered their asthma to be well or completely controlled (Figure 2(e)). Many athletes with well-controlled asthma reported use of rescue medication once or twice daily or 2-3 times per week (Figure 2(d)), whereas the athletes with uncontrolled asthma used SABA at least 2-3 times per week (Figure 2(b)).

The athletes had a significantly higher mean ACT score than the reference subjects (Table 4) but included a smaller proportion of subjects with uncontrolled asthma and a larger proportion of subjects with well-controlled asthma (Table 4). There were no significant sex-related differences in ACT scores within any of the groups. In the multivariate Poisson regression model, there were no associations between ACT score and age, age at asthma onset, allergy, shortness of breath after exercise, or respiratory-related health care contacts during the previous 12 months. However, in the reference group, there was an independent positive association between the ACT score and the age at asthma onset (*p*=0.020) and an independent negative association between the ACT score and exercise-induced shortness of breath (*p*=0.002) and the number of health care contacts (*p*=0.001). When the data for the athletes and reference subjects were pooled, there was no independent association between the study group and ACT score (*p*=0.165).

### 3.3. Asthma Medication

Of the athletes with current asthma, 39% used SABA/LABA daily, 31% used ICS daily, and 31% used ICS + LABA daily. Overall, 43% of the athletes used ICS or ICS + LABA daily. Athletes with an ACT score indicating uncontrolled asthma reported more daily use of *β*2-agonists and ICS + LABA (Table 2). There were no statistically significant differences in the proportions of subjects who used SABA/LABA, ICS, or ICS + LABA daily between the study groups (Table 5). There were also no significant sex-related differences in the use of asthma medication between the groups.

## 4. Discussion

This postal questionnaire survey evaluated disease control and use of asthma medication in 141 elite endurance athletes with asthma and a reference population of 275 nonathletic patients with asthma. Among the elite athletes, 108 (77%) had current asthma, that is, had used asthma medication during the previous 12 months; 39% used SABA/LABA, 31% used ICS, and 31% used ICS + LABA on a regular daily basis. According to the ACT scores, 19% of athletes had current asthma that was uncontrolled, 25% had partly controlled asthma, and 58% had well-controlled asthma. Athletes with uncontrolled asthma reported frequent use of SABA/LABA; after adjustment for confounding factors, their ACT scores and daily use of asthma medication did not differ significantly from the values observed in the reference population of patients with asthma who had never smoked.

To the best of our knowledge, this is the first study to compare asthma control and use of asthma medication between elite athletes with asthma and nonathletic patients with asthma. In order to perform at their best, athletes should not be limited by respiratory problems. This is especially true for those with high ventilation rates, such as endurance athletes. Hence, it was not unexpected that the majority (almost 60%) of the endurance athletes in the present study had well-controlled asthma. However, we were surprised to find that up to one-quarter of the elite endurance athletes were classified as having partly controlled asthma and nearly one-fifth as having uncontrolled asthma, which suggests that it is possible to pursue endurance sports at an elite level without well-controlled asthma. Another explanation could be that ACT is an inadequate tool for assessment of asthma control in elite athletes. Depending on how the athletes interpret the questionnaire, both over- and underestimation of asthma control may occur. Although well validated, ACT may have poor accuracy for assessment of uncontrolled asthma [16], and some of the ACT questions might be poorly adapted for elite athletes. The question “During the past 4 weeks, how often have you had shortness of breath?” might lead to a falsely low score among elite athletes training once or twice a day, whereas “shortness of breath” for a subject with a limited amount of physical activity might be a better indicator of inadequate treatment. In the present study, surprisingly few athletes with current asthma reported shortness of breath after exercise. It is difficult to find publications reporting prevalence of exercise-induced asthma (EIA) among athletes with asthma. Our findings may have several explanations. Firstly, elite athletes with asthma may thoroughly and consistently prevent EIA by pharmacotherapy and consequently not experience shortness of breath after exercise. Secondly, elite endurance athletes training 10–20 hours/week may have a different interpretation of and a high threshold for “shortness of breath post exercise.” Further, the question “How often do you use a *β*2-agonist?” might be misleading because many athletes use these medications before training and/or competitions simply to avoid EIA. SABA may be used in a controlled manner prior to exercise in order to prevent EIA or as unplanned rescue during/after exercise when EIA occurs. From a clinical perspective, the subject/athlete in the latter example could be considered having a poorer level of asthma control. Therefore, athletes might have had misleadingly low ACT scores and subsequently high proportions with uncontrolled and partially controlled asthma.

Several studies have assessed asthma control using ACT in “nonelite athletic” subjects. In those studies, the prevalence of uncontrolled asthma (defined as an ACT score ≤ 19 points) varied in the range of 50%–58% [17–19]. In comparison with those results, both the athletes and the reference subjects in our study appeared to have fairly good asthma control. After adjustments, being an elite athlete was not associated with a higher ACT score. Female sex, early asthma onset, increasing age, and a smoking history are well-known risk factors for uncontrolled asthma [17–19]. The group of athletes in our present study contained a significantly smaller proportion of women, had later asthma onset, and were younger than the reference group, which may explain why being an athlete per se was not associated with better asthma control.

Fifty-two (48%) of the 108 athletes with current asthma in our study used a fixed combination of ICS + LABA sometimes to daily, which is similar to observations in Finnish Olympic athletes reported in 2004 [14]. Direct comparison of the studies is difficult because of differences in the study populations: the Finnish athletes were older, a majority were men, and they trained more. Another study reported on the use of asthma medication by 694 Danish elite athletes who had applied for a therapeutic use exemption for their asthma medication in 2005 [20]. Of 445 athletes who were using asthma medication, 140 (32%) used a fixed combination of ICS + LABA. This finding suggests a trend of increasing use of ICS and/or ICS + LABA among Scandinavian athletes, which may be related to the introduction of the fixed-dose combination inhalers, that is, ICS + LABA [10, 21].

The relatively high use of ICS or ICS + LABA in the present study may also explain why the athletes used less SABA/LABA when compared with Australian summer sports athletes [13]. Of 52 Australian athletes with “ever-doctor diagnosed asthma” who used medication for asthma or asthma-like symptoms, 54% used SABA, 25% used LABA, and 44% used ICS. The lower use of SABA/LABA in the present study could indicate better controlled asthma in Swedish (mostly winter) endurance athletes than in summer sports athletes from Australia or perhaps simply different traditions of asthma pharmacotherapy between the two countries.

Athletes who used SABA/LABA frequently tended to have uncontrolled asthma when classified by their ACT scores. However, frequent use of SABA/LABA contributes to a lower ACT score, which may cause asthma to be classified as partially controlled or uncontrolled. We suggest that this particular group of athletes should receive extra medical attention in order to evaluate and optimize their disease control. However, this study was cross-sectional, so we cannot conclude that frequent use of SABA/LABA is a cause or an effect of uncontrolled asthma. It is in line with our clinical experience that a large proportion of elite endurance athletes are/feel controlled on Step 1 treatment with SABA only and seldom need/use controllers (ICS or ICS/LABA).

In our reference group of patients with asthma who had never smoked, 31% used ICS + LABA daily, which is higher than the rate of 19% reported in a population-based cohort of patients with asthma [10]. Again, direct comparison between the two studies is difficult because our reference group was younger than that in the population-based study, which included patients up to 78 years of age and also smokers.

The present study has some limitations in that the diagnosis of asthma, level of asthma control, and use of asthma medication were based on self-reported data and were not confirmed by medical records or prescriptions of medication. The low response rate of 39% in the reference group may represent a degree of selection bias. A nonresponse analysis would be of value, however, was not included in the original study design and ethical approval. Two Scandinavian questionnaire-based studies found that nonresponders were significantly more likely to be men, younger, and current smokers but found no significant differences with regard to the prevalence of asthma-like symptoms or use of asthma medication [22, 23]. However, an earlier questionnaire-based survey conducted in northern Sweden found that respiratory symptoms and use of asthma medication were more common among nonresponders [24], highlighting the risk of underestimating the prevalence of respiratory symptoms using postal surveys. Our reference group was included based on health care records, so we can assume that they had had a recent consultation for their asthma, that is, during 2011–2015. However, the distribution of asthma control in the reference group is almost identical to the findings from primary care in Sweden 2001 and 2005, a survey with 60% response rate [25]. Altogether, it is possible that the reference group was skewed towards patients with symptomatic asthma and frequent use of asthma medication and thus underestimated ACT scores and overestimated use of asthma medication in this group.

The 66% response rate for the athletes in this study can be considered representative of Swedish endurance athletes, who are competing mostly in a cold climate. Given that the Swedish guideline for asthma treatment follows the international guidelines, the results of our study may be generalizable to endurance athletes outside of Sweden.

It has been shown that German elite athletes with high ventilation rates use significantly more asthma medication than those with medium or low ventilation rates, but no significant difference in use was observed between summer and winter athletes [5]. These observations support our present findings regarding asthma medication and suggest that they may also be applicable to summer endurance athletes, such as long-distance runners and racing cyclists.

## 5. Conclusions

According to our postal questionnaire survey, 39% of Swedish endurance athletes with current asthma used SABA/LABA and 31% used ICS + LABA daily. Most of the athletes had well-controlled asthma (58%), and those with uncontrolled asthma (19%) reported frequent use of SABA/LABA. Their ACT scores and daily use of asthma medication did not significantly differ from the results in the reference population of patients with asthma who had never smoked. Being an elite endurance athlete per se was not associated with either the ACT score or daily use of asthma medication. All patients with asthma should be monitored regularly. Elite endurance athletes using *β*2-agonists several times per week may have uncontrolled asthma and need extra attention in order to minimize the risk of respiratory limitations.

## Figures and Tables

**Table 1:** Demographic and clinical characteristics of a group of Swedish elite endurance athletes and a health care-based reference population of patients with asthma.

	Athletes (*n*=141)	References (*n*=275)	*p* value
Age (years), mean (SD)	21.5 (4.5)	25.6 (6.4)	**<0.001**
Women	79 (56)	181 (66)	0.051
Training (hours/week), mean (SD)	11.7 (4.4)	4.9 (4.5)	**<0.001**
Current asthma	108 (77)	243 (88)	**0.002**
Age of asthma onset (years), mean (SD)	13.3 (4.5)	9.8 (6.7)	**<0.001**
Allergy	54 (50)	203 (74)	**<0.001**
Family history of asthma	78 (59)	149 (58)	0.866
Health care contacts^1^	25 (18)	75 (27)	**0.031**
Shortness of breath^2^	27 (19)	95 (35)	**0.001**

The data are presented as *n* (%) unless otherwise stated; significant *p* values are shown in bold; ^1^respiratory-related health care contacts in the previous 12 months; ^2^shortness of breath following strenuous activity in the previous 12 months; SD, standard deviation.

**Table 2:** Level and distribution of asthma control in Swedish endurance athletes with current asthma.

	Uncontrolled (*n*=19)	Partially controlled (*n*=24)	Well controlled (*n*=59)	*p* value^∗^
Female sex	11 (58)	17 (71)	32 (54)	0.378
Age (years), mean (SD)	19.8 (3.7)	21.3 (4.2)	22.2 (4.8)	0.174
Age of asthma onset (years), mean (SD)	13.4 (4.3)	16.5 (4.0)	12.1 (4.8)	**0.009**
Allergy	9 (47)	14 (58)	23 (44)	0.517
Training (hours/week), mean (SD)	12.9 (2.7)	13.1 (3.8)	11.1 (4.8)	0.855
Shortness of breath^1^	8 (42)	7 (29)	10 (17)	0.079
Health care contacts^2^	9 (47)	8 (33)	7 (12)	**0.003**
Family history of asthma	12 (63)	13 (62)	37 (69)	0.828
Daily SABA/LABA	14 (74)	12 (50)	15 (25)	**<0.001**
Daily ICS	9 (47)	7 (29)	18 (31)	0.353
Daily ICS + LABA	10 (53)	8 (33)	13 (22)	**0.039**

The data are presented as *n* (%) unless otherwise stated; significant *p* values are shown in bold; ^1^shortness of breath following strenuous exercise in the previous 12 months; ^2^respiratory-related health care contacts in the previous 12 months; ICS, inhaled corticosteroids; SABA/LABA, short-acting *β*2-agonists/long-acting *β*2-agonists; SD, standard deviation; ^∗^Pearson's chi-squared test.

**Table 3:** Level and distribution of asthma control in a health care-based reference group of patients with current asthma who have never smoked.

	Uncontrolled (*n*=82)	Partially controlled (*n*=57)	Well controlled (*n*=102)	*p* value^∗^
Females	55 (67)	35 (61)	67 (66)	0.779
Age (years), mean (SD)	24.1 (4.2)	25.6 (4.7)	26.7 (7.8)	**0.006**
Age of asthma onset (years), mean (SD)	7.9 (6.2)	9.7 (7.6)	11.3 (6.3)	**0.003**
Allergy	69 (84)	42 (74)	74 (73)	0.148
Training (hours/week), mean (SD)	5.0 (5.5)	5.3 (4.4)	4.7 (3.7)	0.827
Shortness of breath^1^	48 (59)	21 (38)	24 (24)	**<0.001**
Health care contacts^2^	39 (48)	15 (26)	20 (20)	**<0.001**
Family history of asthma	46 (61)	32 (62)	54 (56)	0.739
Daily SABA/LABA	50 (61)	14 (25)	8 (8)	**<0.001**
Daily ICS	26 (32)	13 (23)	17 (17)	**0.056**
Daily ICS + LABA	36 (44)	13 (23)	38 (37)	**0.037**

The data are presented as *n* (%) unless otherwise stated; significant *p* values are shown in bold; ^1^shortness of breath following strenuous exercise in the previous 12 months; ^2^respiratory-related health care contacts in the previous 12 months; ICS, inhaled corticosteroids; SABA/LABA, short-acting *β*2-agonists/long-acting *β*2-agonists; SD, standard deviation; ^∗^Pearson's chi-squared test.

**Table 4:** Bivariate analyses comparing ACT scores and levels of the control based on ACT scores between Swedish elite endurance athletes and a reference group of patients with current asthma who have never smoked.

	Athletes (*n*=108)	References (*n*=242)	*p* value
ACT score, mean (SD)	22.2 (2.9)	21.0 (4.0)	**0.004**
Uncontrolled^1^	19 (19)	81 (34)	**0.005**
Partially controlled^2^	24 (24)	57 (23)	0.965
Well controlled^3^	59 (58)	102 (42)	**0.009**

The data are presented as *n* (%); significant *p* values are shown in bold; ^1^ACT score ≤ 19; ^2^ACT score 20–21; ^3^ACT score ≥ 22; ACT, Asthma Control Test; SD, standard deviation.

**Table 5:** Use of asthma medication by Swedish elite endurance athletes and a reference group of patients with current asthma and a statistical comparison between the two groups.

	Athletes (*n*=108)	References (*n*=242)	*p* value^∗^
SABA/LABA			
Never	32 (30)	40 (17)	
Sometimes	30 (28)	101 (42)	
Less than 2 months	0 (0)	12 (5)	
More than 2 months	4 (4)	16 (7)	
Daily	42 (39)	73 (30)	0.101
ICS			
Never	56 (52)	135 (56)	
Sometimes	14 (13)	34 (14)	
Less than 2 months	1 (1)	8 (3)	
More than 2 months	3 (3)	9 (4)	
Daily	34 (31)	56 (23)	0.099
ICS + LABA			
Never	64 (59)	126 (52)	
Sometimes	5 (5)	17 (7)	
Less than 2 months	3 (3)	5 (2)	
More than 2 months	3 (3)	7 (3)	
Daily	33 (31)	87 (36)	0.326

To compare the two groups with regard to use of asthma medication, the subjects were dichotomized into those who did and did not use SABA/LABA, ICS, or ICS + LABA daily; ^∗^comparison of proportions of daily users of SABA/LABA, ICS, and ICS + LABA between the study groups; ICS, inhaled corticosteroids; SABA/LABA, short-acting *β*2-agonists/long-acting *β*2-agonists.
